# Supplementary material for: Visualization of Sirtuin 4 Distribution between Mitochondria and the Nucleus, Based on Bimolecular Fluorescence Self-Complementation
Source: Cells. 2019 Dec 6;8(12):1583. doi: 10.3390/cells8121583 (PMC6953047; doi:10.3390/cells8121583)
Supplement: Supplementary file 1 [file cells-08-01583-s001.pdf]

Visualization of Sirtuin 4 Distribution between Mitochondria and the Nucleus, Based on Bimolecular Fluorescence Self-Complementation

Jeta Ramadani-Muja [1], Benjamin Gottschalk [1], Katharina Pfeil [2], Sandra Burgstaller [1], Thomas Rauter [1], Helmut Bischof [1], Markus Waldeck-Weiermair [1], Heiko Bugger [2], Wolfgang F. Graier [1,3] and Roland Malli [1,3,*]

[1] Gottfried Schatz Research Center, Chair of Molecular Biology and Biochemistry, Medical University of Graz, Neue Stiftingtalstraße 6/6, 8010 Graz, Austria; jeta.ramadani@medunigraz.at (J.R.-M.); benjamin.gottschalk@medunigraz.at (B.G.); sandra.burgstaller@medunigraz.at (S.B.); thomas.rauter@medunigraz.at (T.R.); helmut.bischof@medunigraz.at (H.B.); markus.weiermair@medunigraz.at (M.W.-W.); wolfgang.graier@medunigraz.at (W.F.G.);

[2] Division of Cardiology, Medical University of Graz, Graz, Austria; katharina.pfeil@medunigraz.at (K.P.); heiko.bugger@medunigraz.at (H.B)

[3] BioTechMed Graz, Mozartgasse 12/II, 8010 Graz, Austria

* Correspondence: roland.malli@medunigraz.at; Tel.: +43-(0)-316-385-71956

Supplementary Figure S1

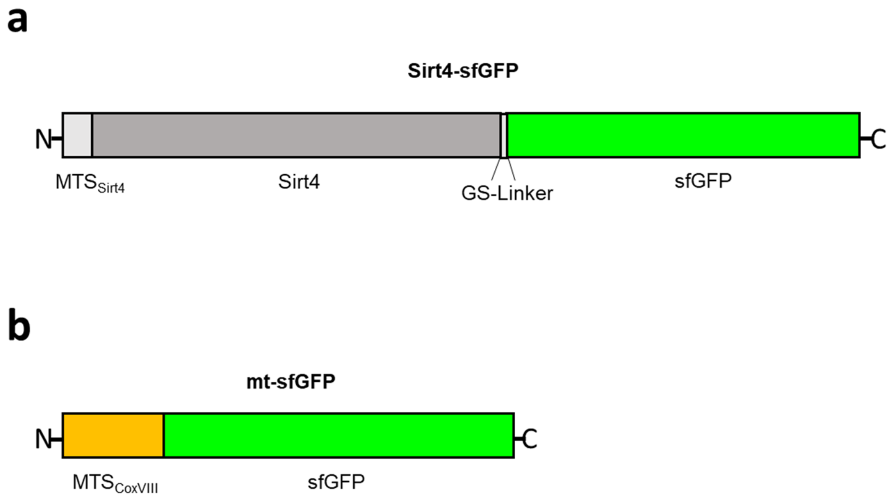

Supplementary Figure S1. Schematic overview of the Sirt4-sfGFP and mt-sfGFP. (a) Mitochondrial targeting sequence of the Sirt4 shown in light grey, referred to as MTS_{Sirt4} followed by the rest of the Sirt4 illustrated in dark grey. Sirt4 is C-terminally fused to the sfGFP, shown in bright green. (b) Mitochondrial targeting sequence of the CoxVIII subunit, referred to as $MTS_{CoxVIII}$ (orange) fused on N-terminus of the sfGFP.

Supplementary Figure S2

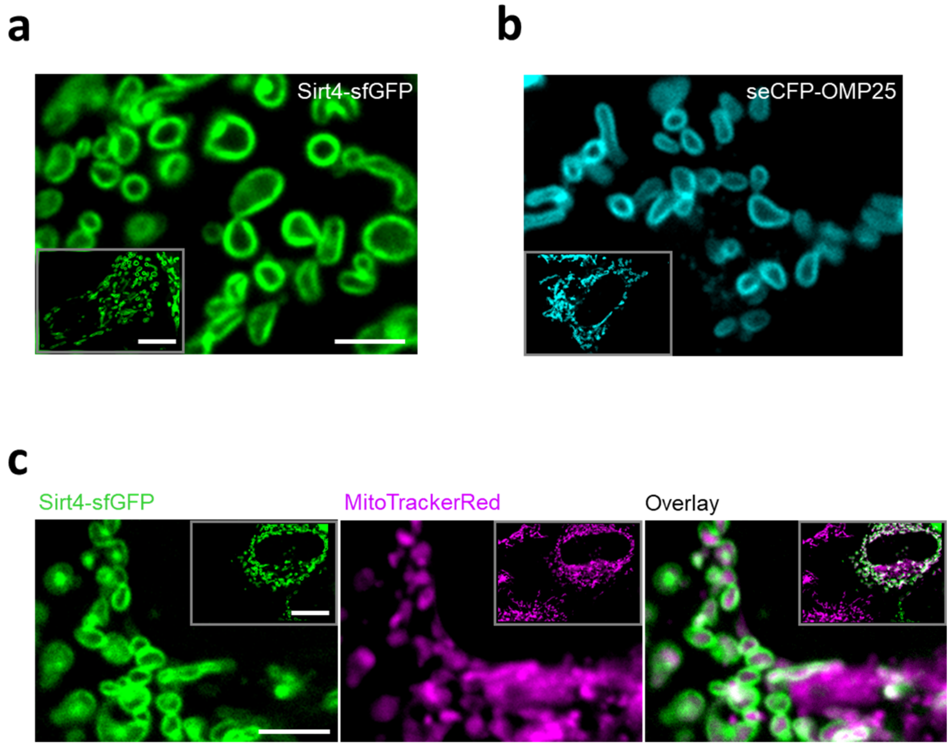

Supplementary Figure S2. Representative confocal images of either Sirt4-sfGFP or seCFP-OMP25 and colocalization images of Sirt4-sfGFP with MitoTrackerRed. (a) HeLa cells expressing Sirt-sfGFP. Scale bar represents 2.5 µm. Square in the lower left shows the whole cell. Scale bar represents 10 µm. (b) Identical experiment as this in a, but using seCFP-OMP25. (c) Representative confocal images of HeLa cells expressing Sirt4-sfGFP (green) and stained with MitoTrackerRed® (magenta). Right panel represents merged image. Scale bar indicating 2.5 µm. Square in the upper right illustrates the whole cell. Scale bar represents 10 µm. Pearson correlation coefficient: 0.28 (mean) with an standard deviation of 0.14. Shown experiments are representative of 3 independent experiments, encompassing 30 different cells.

Supplementary Figure S3

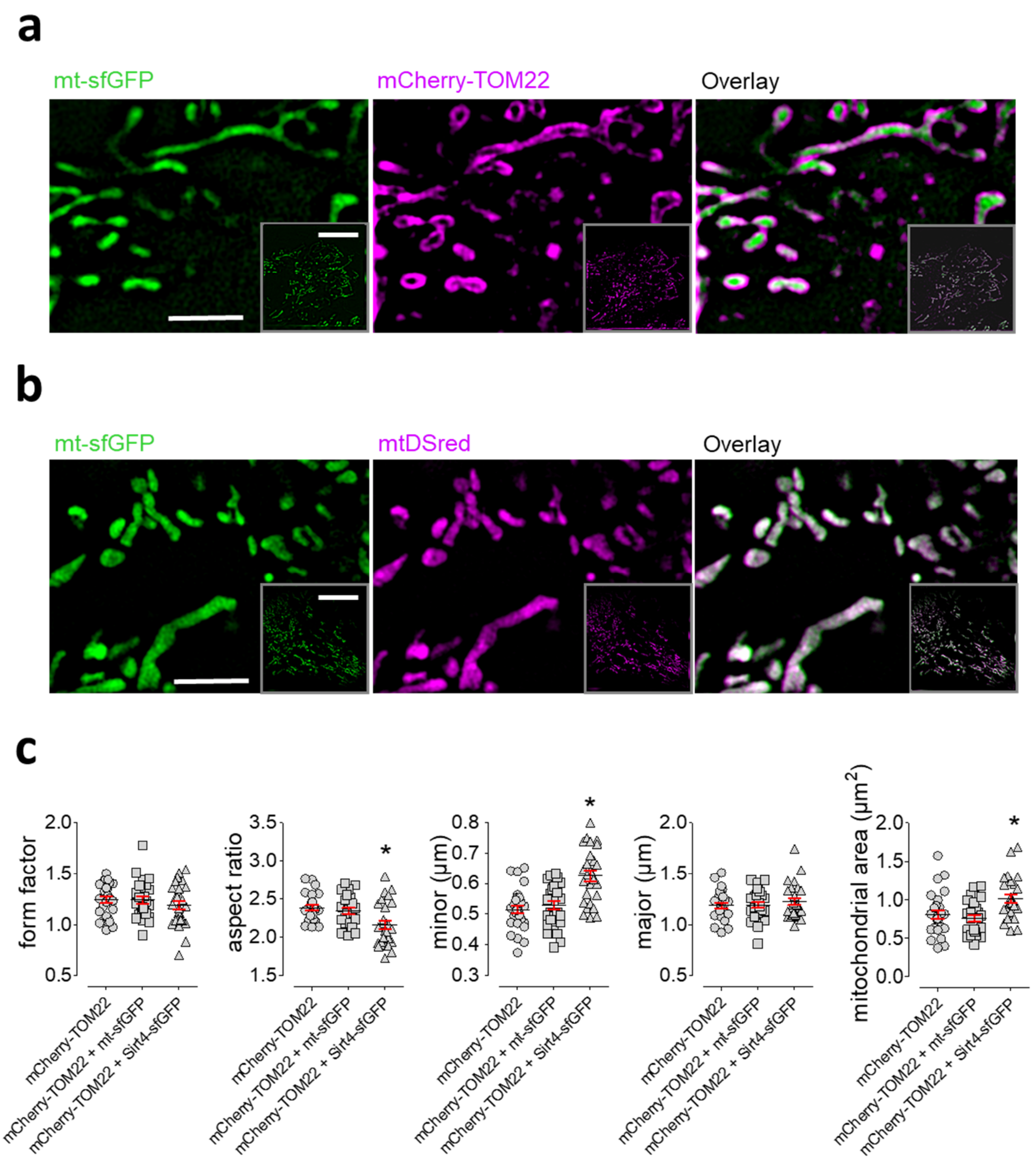

Supplementary Figure S3. Co-localization of Sirt4-sfGFP and mCherry-TOM22 and mtDSred, respectively (a) Representative SIM-images of HeLa cells co-expressing Sirt-sfGFP (green) and mCherry-TOM22 (magenta). Illustration of merged channels. Scale bar, 2.5 µm. Pearson correlation coefficient: 0.58 (mean) with an standard deviation of 0.09. Squares in the lower right show the whole cell. Scale bar represents 10 µm. (b) Identical experiment as this in a, but using mtDSred. Pearson correlation coefficient: 0.92 (mean) with an standard deviation of 0.02. Shown experiments in a and b are representative of 3 independent experiments, including 30 different cells (c) Analysis of mitochondrial morphology. First diagram represents values of the form factor. Second diagram shows the aspect ratio. Third and fourth diagram illustrate the minor and major values, respectively. The last diagram represents the values of the mitochondrial area. For all conditions, cells are represented in grey shapes, respectively. Data are shown as the mean ± SEM; $n = 30$ cells. For testing significance ANOVA with Bonferroni post-hoc test was used. (* $p < 0.05$).

Supplementary Figure S4

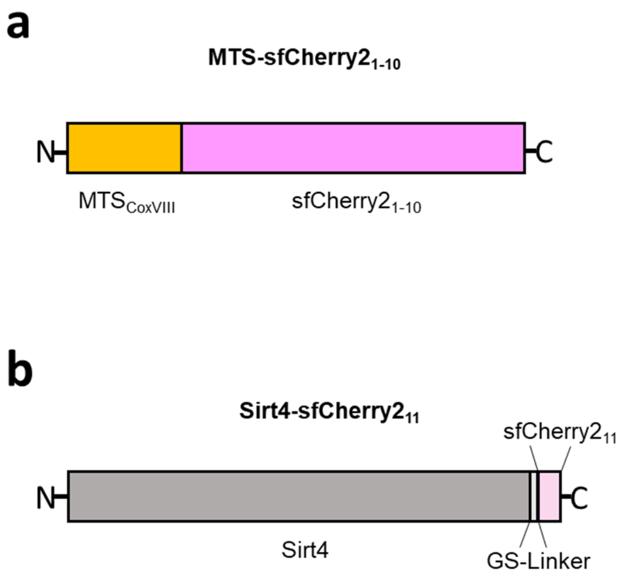

Supplementary Figure 4. Scematic illustration of the probes, based on the self-complementing split-FP variant of the sfCherry2. (a) Mitochondrial targeting sequence of the CoxVIII subunit, referred to as MTS$_{CoxVIII}$, depicted in orange fused C-terminally to the sfCherry2$_{1-10}$, here shown in pink (b) Sirt4, illustrated in dark grey, fused C-terminally to the sfCherry2$_{11}$.

Supplementary Figure S5

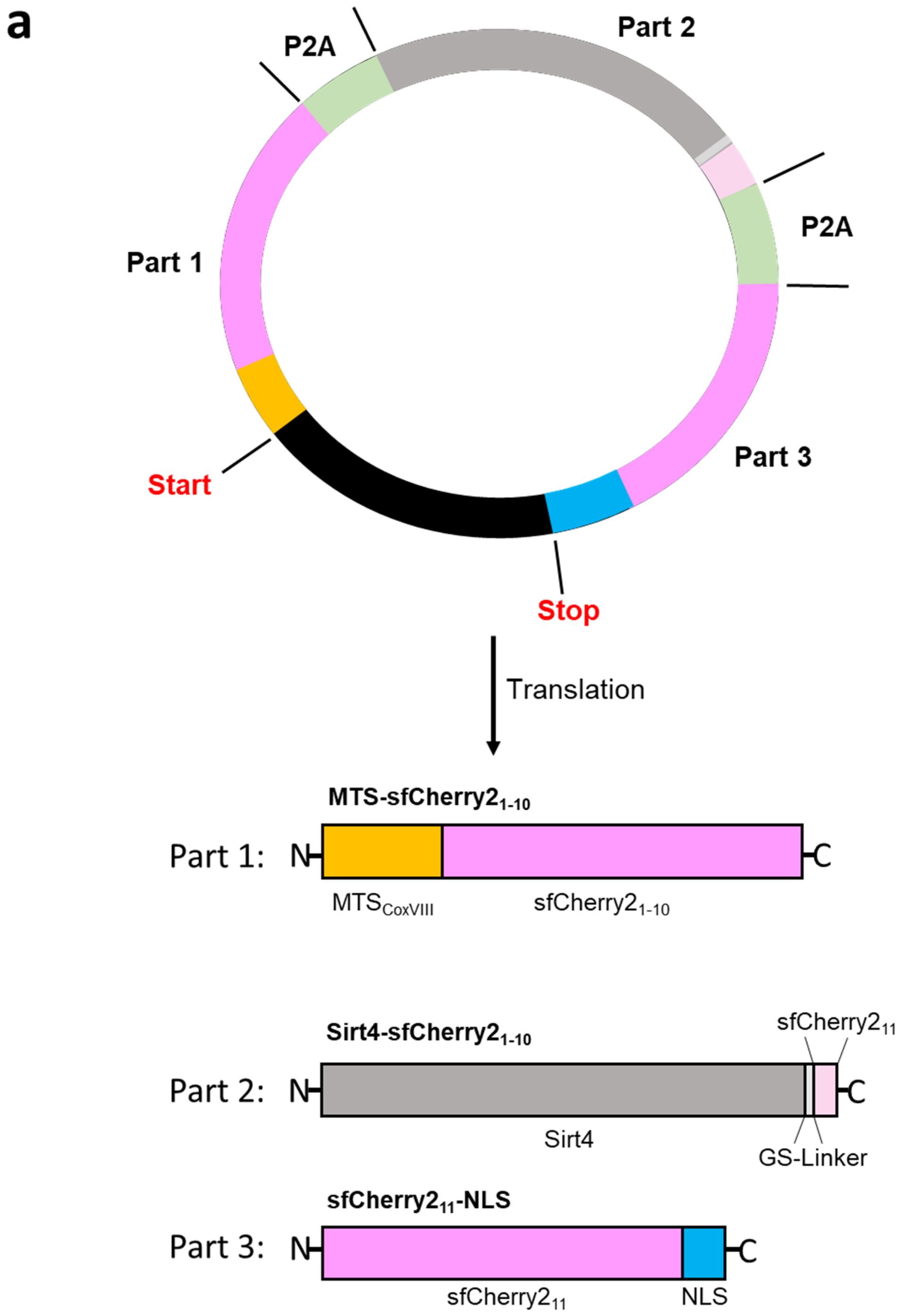

Supplementary Figure 5. Overview of the mito-STAR construction. (a) Scematic illustration of the plasmid coding for the sensor mito-STAR, including three parts (Part 1: MTS-sfCherry2$_{1-10}$, part 2: Sirt4-sfCherry2$_{1-10}$ and part 3: sfCherry2$_{11}$-NLS), inbetween harboring a peptide with self-cleavage acitiviy (green), depicted as P2A. Translation of the probe results in three differentially targeted individual parts. Part 1 representing MTS-sfCherry2$_{1-10}$. Part 2 illustrates Sirt4-sfCherry2$_{1-10}$. Part 3 shows sfCherry2$_{11}$-NLS.

Supplementary Figure S6

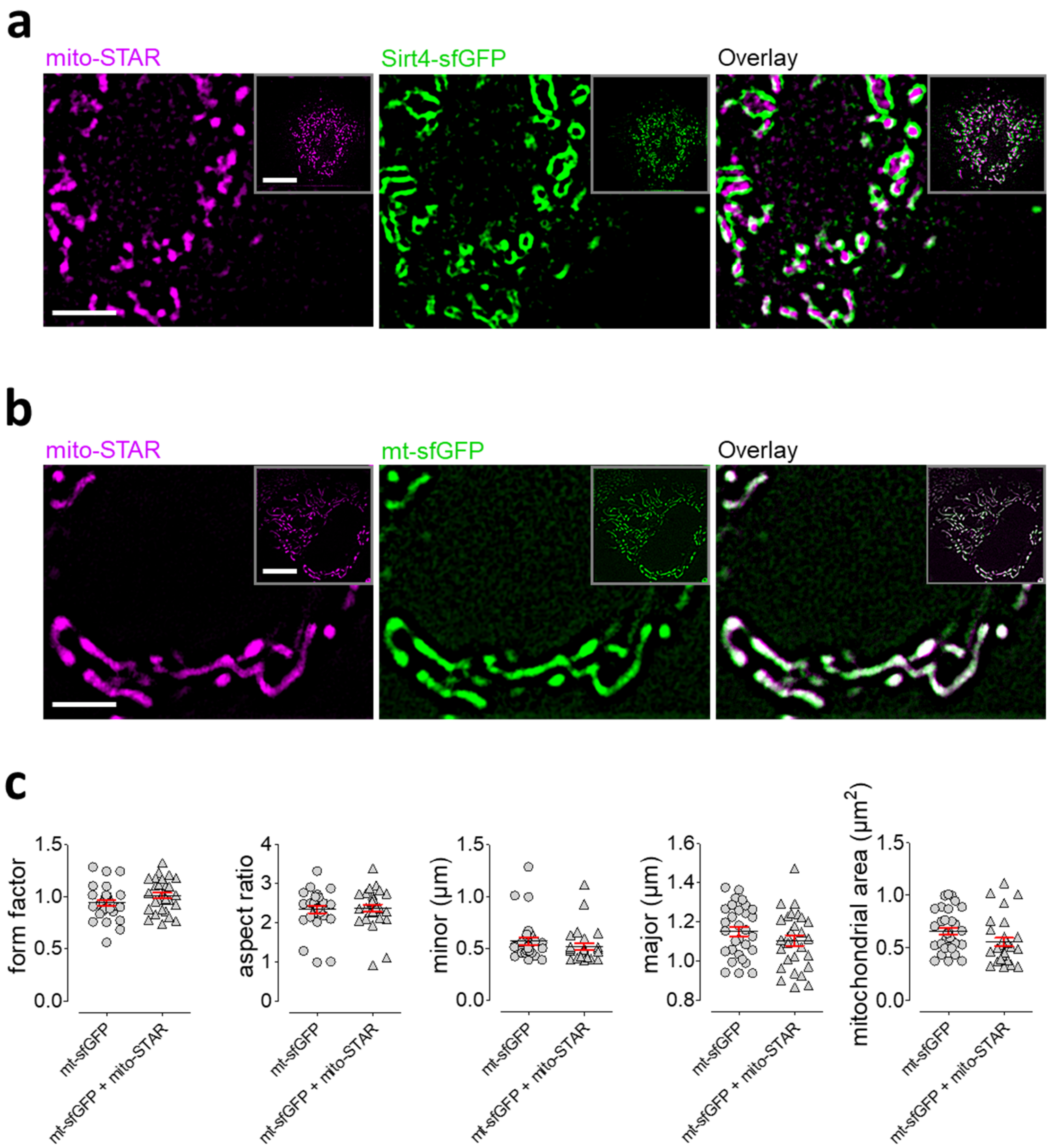

Supplementary Figure 6. Co-localization of mito-STAR and mCherry-TOM22 and mtDSred, respectively proving the import of Sirt4-sfCherry2$_{11}$ into the lumen of mitochondria. (a) Representative SIM-images of HeLa cells co-expressing mt-sfGFP (green) and mCherry-TOM22 (magenta). Illustration of merged channels as an overlay image. Scale bar, 2.5 µm. Squares in the lower right show the whole cell. Scale bar represents 10 µm. Pearson correlation coefficient: 0.39 (mean) with an standard deviation of 0.11. (b) Identical experiment as this in a, but using mtDSred. Pearson correlation coefficient: 0.85 (mean) with an standard deviation of 0.06. Shown experiments in a and b are representative of 3 independent experiments, including 30 different cells. (c) Analysis of mitochondrial morphology. First diagram represents values of the form factor. Second diagram shows the aspect ratio. Third and fourth diagram illustrate the minor and major values, respectively. The last diagram represents the values of the mitochondrial area. For all conditions, cells are represented in grey shapes, respectively. Data are shown as the mean ± SEM; n = 30 cells. For testing significance unpaired double sided *t-test* was used.
